# *Sophora japonica* Extract Supplementation Facilitates Piglets’ Early Weaning Transition by Alleviating Intestinal Oxidative Stress, Inflammation, and Microbiota Dysbiosis

**DOI:** 10.3390/vetsci13090887

**Published:** 2026-08-30

**Authors:** Ting Yang, Wenxia Qin, Yunzheng Xu, Yuwen Chen, Juncheng Huang, Erdan Wang, Libao Ma, Caimei Yang, Baoyang Xu

**Affiliations:** 1 College of Animal Science and Technology, College of Veterinary Medicine, Zhejiang A&F University, Hangzhou 311300, China; yangtingly@126.com (T.Y.);; 2 College of Animal Science and Technology, Huazhong Agricultural University, Wuhan 430070, China

**Keywords:** *Sophora japonica* extract, oxidative stress, inflammation, microbiota dysbiosis, early-weaned piglets

## Abstract

The early weaning of piglets is still widely practiced in modern swine production, although it is frequently associated with intestinal dysfunction, microbiota dysbiosis, and even diarrhea. A total of 126 piglets were randomly allotted to three groups (seven pens per group) and fed either a basal diet, a basal diet supplemented with 0.05% SJE, or a basal diet supplemented with 0.1% SJE. Supplementation with 0.1% SJE effectively decreased fecal scores on days 7 and 14 post-weaning and improved growth performance on day 14 post-weaning in early-weaned piglets. In addition, SJE supplementation improved jejunal morphology, increased antioxidant capacity indices, and reduced oxidative metabolites in jejunal tissue. Correspondingly, SJE supplementation upregulated the mRNA expression of antioxidant-related genes and anti-inflammatory cytokines in the jejunum. Notably, SJE supplementation altered the gut microbiota structure and increased alpha diversity indices, including observed species, ACE, and Chao1. Specifically, SJE supplementation increased the relative abundances of Fibrobacteres, *Akkermansia muciniphila*, and *Prevotella copri*. The SJE-induced shifts in gut microbiota were signific antly correlated with early weaning–related indices. Overall, SJE supplementation alleviated intestinal oxidative stress, inflammation, and microbiota dysbiosis, which may contribute to its effects on alleviating diarrhea, intestinal oxidative stress, inflammation, and microbiota dysbiosis in early-weaned piglets.

## Simple Summary

The early weaning of piglets remains widely practiced in intensive swine production, although it is frequently associated with intestinal oxidative stress, inflammation, and microbiota dysbiosis. In this study, we evaluated the effects of *Sophora japonica* extract (SJE) on attenuating intestinal oxidative stress, inflammation, and microbiota dysbiosis in early-weaned piglets. Supplementation with 0.1% SJE significantly reduced fecal scores on days 7 and 14 post-weaning, while also improving growth performance during days 8–14 post-weaning in early-weaned piglets. Moreover, SJE supplementation enhanced jejunal morphology, elevated intestinal antioxidant enzyme activities, and concurrently decreased oxidative metabolite levels. Consistently, SJE supplementation upregulated the expression of antioxidant-related genes in the jejunum. Additionally, SJE supplementation increased the expression of anti-inflammatory cytokines. Notably, SJE supplementation also reshaped the gut microbiota and increased alpha diversity indices, including observed species, ACE, and Chao1. The SJE-induced changes in gut microbiota were significantly correlated with early weaning–related indices. These findings provide evidence that SJE may be used to alleviate diarrhea, intestinal oxidative stress, inflammation, and microbiota dysbiosis in early-weaned piglets.

## 1. Introduction

Piglets are commonly weaned at three to four weeks of age in modern swine production systems to improve efficiency, whereas natural weaning would occur at approximately 17 weeks after birth [1,2,3]. As a result, early-weaned piglets often experience transient anorexia, severe intestinal damage, intestinal oxidative stress, intestinal inflammation, enteral infections, and diarrhea due to changes in diet as well as the social and environmental conditions [4,5]. To manage early-weaning stress, conventional practices have heavily relied on supplementing diets with pharmacological levels of zinc and copper, along with the widespread use of antimicrobial agents [2,3]. However, these strategies have drawn growing concern due to their contribution to environmental pollution and the accelerating threat of antibiotic resistance [2,3]. With the expansion of the ban on the use of antibiotic growth promoters for disease prevention and growth promotion in swine production, as well as restrictions on copper and zinc additions, there is an urgent need to identify replacement candidates for antibiotics capable of alleviating early-weaning stress [2,6,7,8,9]. Increasing evidence has indicated that gut microbiota dysbiosis in early-weaned piglets is a possible contributor to both post-weaning scours and intestinal infectious conditions [2,10]. Specifically, intestinal barrier damage and inflammation lead to oxidative stress, which further disturbs the oxygen-sensitive niches occupied by gut microbiota, thereby inducing dysbiosis [2,11]. Therefore, non-antibiotic strategies capable of restoring intestinal redox homeostasis are potentially effective agents for facilitating the early-weaning transition in piglets [2].

Plant-derived antioxidant substances, especially polyphenolic compounds, are capable of eliminating free radicals and ameliorating intestinal dysfunction triggered by oxidative stress [12]. *Sophora japonica* extract (SJE) is rich in flavonoid polyphenols that are commonly found in vegetables, fruits, and Chinese herbs [13]. Enriched with multiple bioactive flavonoids, SJE has long been applied in traditional Asian medical systems (including Korean medicine) for its diverse therapeutic values [14]. The principal components of SJE include kaempferol, quercetin, and rutin [15]. A growing body of recent in vitro and in vivo studies has verified that SJE possesses extensive biological properties, encompassing anti-inflammatory, bacteriostatic, antiviral, anti-osteoporosis, antioxidative, free-radical elimination, hypoglycemic, anti-obesity, anti-tumor, and hemostatic activities [15,16,17,18,19]. In addition, animal experiments using a castor oil–induced rat diarrhea model have demonstrated that SJE can effectively reduce the total output and wet weight of feces in rats with diarrhea in a dose-dependent manner [17]. Collectively, these findings indicate that SJE is a high-value plant extract with great potential for developing novel anti-diarrheal additives applicable to early-weaned piglets.

Accordingly, this study proposed a research hypothesis that dietary supplementation with SJE could alleviate intestinal damage and restore redox homeostasis in early-weaned piglets, and that gut microbiota may serve as a key pharmacological target for SJE to exert its regulatory effects. To verify this hypothesis, two supplemental doses of SJE (0.05% and 0.1%) were applied to early-weaned piglets, and the regulatory effects of SJE on intestinal damage, redox disorder, and gut microbial dysbiosis in piglets were comprehensively investigated.

## 2. Materials and Methods

### 2.1. Experimental Statement

All procedures involving animals were reviewed and approved by the Animal Experimental Ethical Inspection Committee of the Laboratory Animal Center at Huazhong Agricultural University (Approval number: HZAUSW20210012; ethics approval date: 22 October 2021).

### 2.2. Materials and Chemicals

The SJE used in this study had a purity of 95.18% and was extracted from *Sophora japonica* (Hengrui Tongda Bio. Co. Ltd., Chengdu, China). SJE was extracted following the *Veterinary Pharmacopoeia of the People’s Republic of China (2015 edition)*. The SJE used in this study contains a purity of 95.18%, with the major bioactive flavonoids being rutin (48.2%), quercetin (12.5%), and kaempferol (8.3%), as determined by high-performance liquid chromatography U-3000 (Thermo Scientific, Waltham, MA, USA) using authentic reference standards.

### 2.3. Animals and Experimental Treatments

A total of 126 early-weaned Landrace × Yorkshire piglets (23 ± 1 d of age, with equal numbers of males and females) were randomly allotted to three groups (7 pens per group, 6 piglets per pen) and fed either a basal diet (Ctrl), a basal diet supplemented with 0.05% SJE (0.05% SJE), or basal diet supplemented with 0.1% SJE (0.1% SJE) from day 0 to day 14 post-weaning (temperature: 28–32 °C; humidity: 60~80%). During the experiment, the early-weaned piglets in each group were free to eat and drink. Piglets were vaccinated against porcine circovirus at around 7 days of age and against porcine mycoplasma pneumonia (respiratory disease) at 14 days of age. The basal diet was formulated according to the recommendations of the National Research Standards Committee (NRC, 2012) to meet the nutrient requirements of pigs (Table S1), and the supplementation doses of SJE used in this study were determined based on prior relevant studies [17].

### 2.4. Growth Performance and Fecal Scores

The average daily gain (ADG) and average daily feed intake (ADFI) were determined per pen to evaluate the growth performance of piglets. Fecal scores were used to determine piglets’ early-weaning transition. Fecal scores were assessed per pen daily, and only diarrhea data from days 7 and 14 were analyzed using a four-point scoring system, ranging from 1 (normal, well-formed feces) to 4 (severe, watery diarrhea), as previously described [20,21]. To reduce subjective error, the fecal score of piglets was calculated by averaging the scores from three independent observers.

### 2.5. Sample Collection

On day 14 post-weaning, based on the observed effects of 0.05% and 0.1% SJE supplementation on fecal scores, the control (Ctrl) and 0.1% SJE (SJE) groups were selected for the slaughter experiment. One piglet per replicate from each of these two groups was then randomly chosen for sample collection. Euthanasia of the piglets was performed through intravenous injection with sodium pentobarbital, and the applied dose was 50 mg/kg body weight. Three 3-centimeter-long segments were removed from the middle locations of the small intestine; one was placed in 4% paraformaldehyde for histological evaluation, and the other two segments were stored at −80 °C after freezing in liquid nitrogen. After homogenization, colonic contents were collected and then stored at −80 °C after freezing in liquid nitrogen.

### 2.6. Histomorphology Examination of the Jejunal Tissue

To preserve the jejunal samples, the tissue was fixed overnight in 4% paraformaldehyde at 4 °C. Following fixation, the specimens underwent graded ethanol dehydration (70–100%) and were subsequently embedded in paraffin wax. The resulting blocks were cut into 7 μm sections, which were mounted onto glass slides and incubated at 56 °C for 24 h to ensure firm adhesion. After dewaxing and rehydration, the slides were stained with hematoxylin and eosin. Morphological evaluation was carried out using an Olympus BX51 microscope equipped with a digital imaging system (Olympus Co., Tokyo, Japan). Villus height was measured as the straight-line span from the crypt–villus junction to the villus tip, while crypt depth was recorded as the distance from the crypt base to the junction at the crypt opening.

### 2.7. Antioxidant Capacity Evaluation of the Jejunal Tissue

The antioxidant capacity indices of piglets’ jejunal tissue, including total antioxidant capacity (T-AOC), catalase (CAT), malondialdehyde (MDA), glutathione (GSH)/oxidized glutathione disulfide (GSSG), and nitric oxide (NO), were determined using commercial kits from Solarbio Science & Technology (Solarbio Science & Technology, Beijing, China) according to the manufacturer’s instructions, respectively. The GSH/GSSG ratio was calculated by first measuring GSH and GSSG concentrations separately (using the respective kit protocols) and then dividing the GSH value by the GSSG value.

### 2.8. mRNA Expression of Inflammation and Antioxidant Capacity–Related Genes in Jejunal Tissue

Total RNA was isolated from jejunal tissue using TRIzol reagent (Invitrogen, Carlsbad, CA, USA), and its purity and concentration were assessed with a NanoDrop 2000 spectrophotometer (Thermo Scientific, Waltham, MA, USA). Subsequently, 2 µg of the extracted RNA served as the template for first-strand cDNA synthesis, which was carried out with a reverse transcription kit (Thermo Scientific, Waltham, MA, USA) following the supplier’s recommended protocol. Quantitative real-time PCR was then conducted on a CFX384 system (Bio-Rad, Hercules, CA, USA) using iTaq Universal SYBR Green Super Mix (Bio-Rad, Hercules, CA, USA). The thermal cycling conditions included initial denaturation at 95 °C for 5 min, followed by 40 cycles of 95 °C for 15 s, 60 °C for 30 s, and 72 °C for 30 s, with a final elongation step at 72 °C. Relative transcript levels were calculated using the 2^−ΔΔCt^ method and normalized against the endogenous reference gene GAPDH. Relative transcript abundances were normalized to the internal reference gene GAPDH. All primer sequences are provided in Table S2.

### 2.9. Gut Microbial DNA Extraction and Sequencing

Microbial genomic DNA was extracted from colonic content samples using the Stool DNA Kit (TIANGEN, Beijing, China). The V4 hypervariable region of the bacterial 16S rRNA gene was amplified via PCR with the universal primer pair 515F (5′-GTGYCAGCMGCCGCGGTAA-3′) and 806R (5′-GGACTACNVGGGTWTCTAAT-3′). Following purification, the resulting amplicons were pooled in equal molar ratios and subjected to paired-end sequencing (2 × 250 bp) on a NovaSeq 6000 platform (Illumina, San Diego, CA, USA) at Biomarker Technologies Co., Ltd. (Beijing, China).

### 2.10. Gut Microbial Data Analysis

Raw sequencing reads were processed using the QIIME2 pipeline (v.2021.6) for initial quality control and feature table construction [22]. USEARCH (v.10.0) was then applied to cluster high-quality sequences into operational taxonomic units (OTUs) at a 97% similarity threshold, followed by chimera removal using UCHIME (version 8.1) [23,24]. Taxonomic assignment for each OTU representative sequence was performed using the RDP Classifier, with reference to the SILVA (Release 132) and Greengenes (v.13.5) databases [25,26]. Alpha diversity metrics, including observed species, Chao1, Abundance-Based Coverage Estimator (ACE), Shannon, and Simpson indices, were computed using Mothur (v.1.31.2) [27]. Beta diversity was assessed by principal coordinate analysis (PCoA) based on Bray–Curtis dissimilarities, and statistical differences among groups were tested using permutational multivariate analysis of variance (PERMANOVA) with 999 Monte Carlo permutations, both implemented via the ‘vegan’ package in R (v.4.3.1) [21,28].

### 2.11. Data Processing and Statistical Analysis

Statistical analyses were performed using GraphPad 8.0 software. Comparisons between two groups were conducted using two-sided Student’s t-tests, while comparisons among multiple groups were analyzed using ANOVA followed by Duncan’s multiple comparison test. For the fecal scores, non-parametric tests were used (Kruskal–Wallis followed by Mann–Whitney U). Results are presented as mean ± SD, and significance levels are indicated as * *p* < 0.05, ** *p* < 0.01, and *** *p* < 0.001.

## 3. Results

### 3.1. SJE Supplementation Attenuated Diarrhea in Early-Weaned Piglets

As shown in Table 1, compared with the Ctrl group, 0.1% SJE significantly reduced fecal scores on days *7* and 14 post-weaning (*p* < 0.05). Therefore, 0.1% SJE was identified as an effective dose and was designated as the SJE treatment group (SJE) for subsequent analysis.

### 3.2. SJE Supplementation Improved Growth Performance in Early-Weaned Piglets

As shown in Table 2, compared with the Ctrl group, SJE supplementation significantly decreased the F/G ratio on days 7 and 14 (*p* < 0.05) and significantly increased BW (*p* < 0.05), ADFI (*p* < 0.05), and ADG (*p* < 0.01) on day 14.

### 3.3. SJE Supplementation Improved Intestinal Morphology in Early-Weaned Piglets

**Figure 1:**
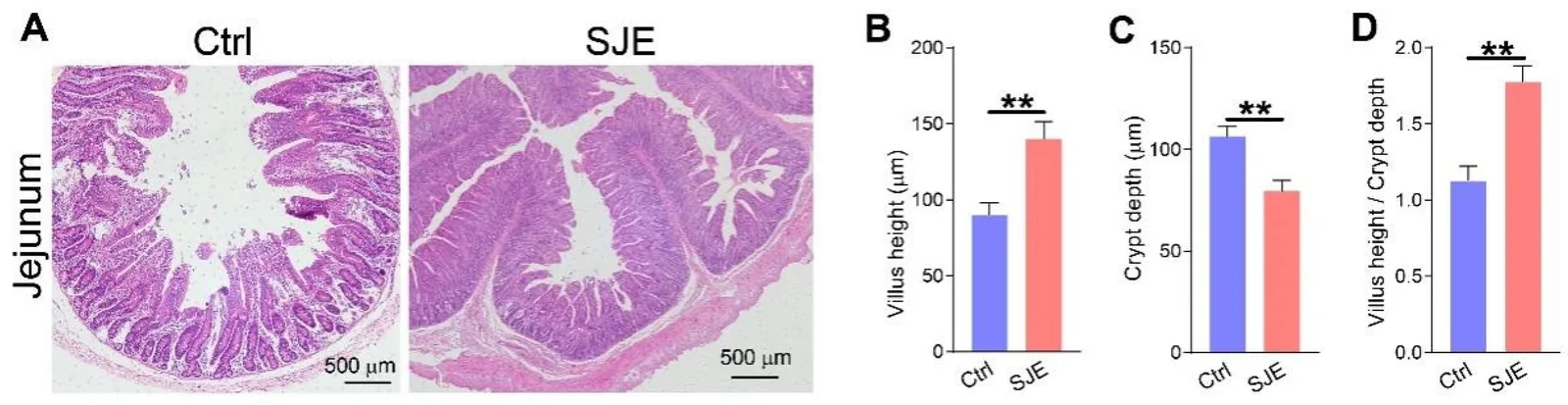
The effect of SJE supplementation on jejunal morphology in early-weaned piglets: (**A**) Representative H&E-stained jejunum sections, (**B**) villus height, (**C**) crypt depth, and (**D**) villus height/crypt depth ratio of the jejunum. SJE, *Sophora japonica* extract; Ctrl group, piglets fed the basal diet; SJE group, piglets fed the basal diet supplemented with 0.1% SJE. Data are presented as mean ± SD (*n* = 7); significance is shown as ** *p* < 0.01.

The intestinal morphology of early-weaned piglets is presented in Figure 1. Compared with the Ctrl group, SJE supplementation improved jejunal morphology (Figure 1A), as indicated by an increased villus height (*p* < 0.01), an increased villus height/crypt depth ratio (*p* < 0.01) (Figure 1B,D), and decreased crypt depth (*p* < 0.01) (Figure 1C).

### 3.4. SJE Supplementation Improved Intestinal Redox Imbalance in Early-Weaned Piglets

**Figure 2:**
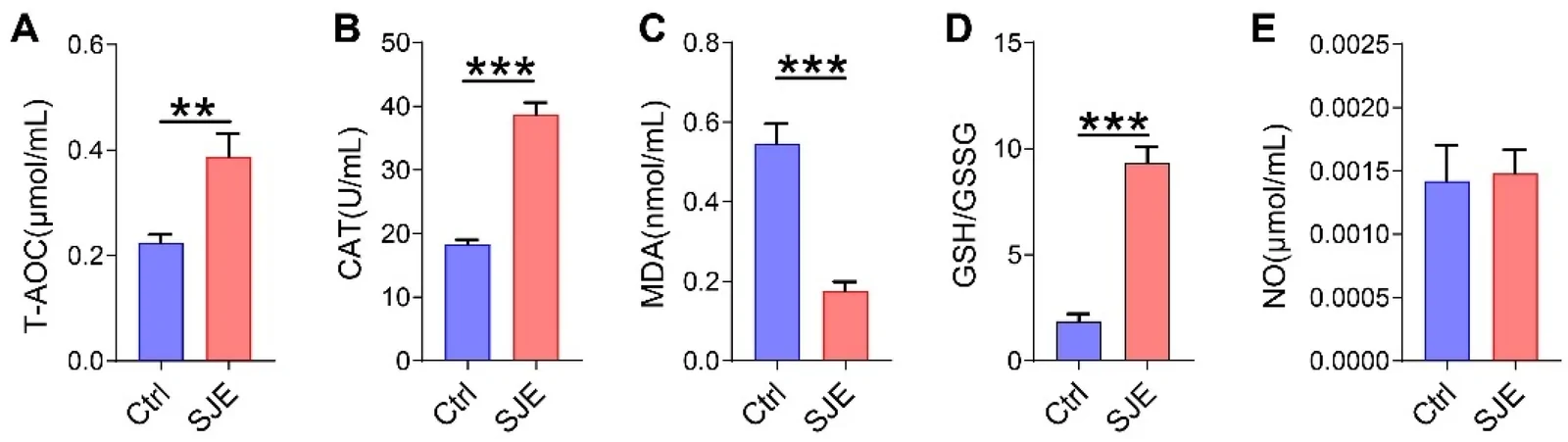
Effects of SJE supplementation on oxidative stress indices in the jejunum of early-weaned piglets. Oxidative stress indices include (**A**) T-AOC, (**B**) CAT, (**C**) MDA, (**D**) the GSH/GSSG ratio, and (**E**) NO. T-AOC, total antioxidant capacity; GSH, glutathione; GSSG, glutathione disulfide; MDA, malondialdehyde; CAT, catalase; NO, nitric oxide; SJE, *Sophora japonica* extract; Ctrl group, piglets fed the basal diet; SJE group, piglets fed the basal diet supplemented with 0.1% SJE. Data are presented as mean ± SD (*n* = 7). Significance is shown as ** *p* < 0.01 and *** *p* < 0.001.

The effects of SJE supplementation on intestinal redox imbalance in early-weaned piglets are shown in Figure 2. Compared with the Ctrl group, SJE supplementation significantly increased antioxidant indices, including T-AOC (Figure 2A, *p* < 0.01), CAT (Figure 2B, *p* < 0.001), and the GSH/GSSG ratio (Figure 2D, *p* < 0.001), while it significantly reduced the oxidative metabolite MDA (Figure 2C, *p* < 0.001) in jejunal tissue. SJE supplementation had no significant effect on NO levels (Figure 2E). These results indicate that SJE supplementation improved intestinal redox imbalance in early-weaned piglets.

### 3.5. SJE Supplementation Regulated Intestinal Gene Expression of Inflammatory Cytokines and Antioxidant-Related Genes in Early-Weaned Piglets

**Figure 3:**
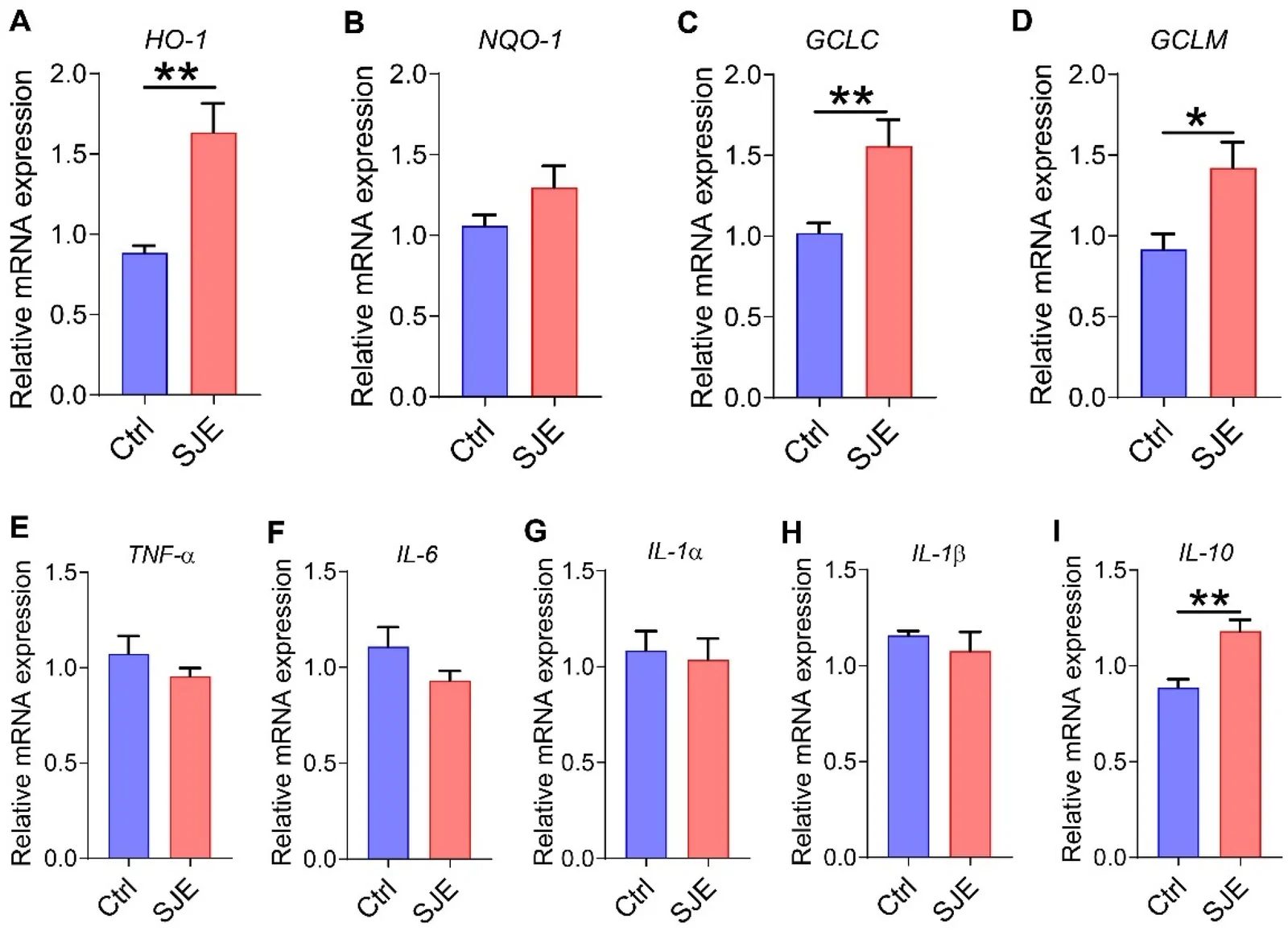
Effects of SJE supplementation on the mRNA expression of inflammatory cytokines and antioxidant-related genes in early-weaned piglets. Antioxidant-related genes: (**A**) *HO-1*, (**B**) *NQO-1*, (**C**) *GCLC*, and (**D**) *GCLM*; inflammatory cytokine–related genes: (**E**) *TNF-α*, (**F**) *IL-6*, (**G**) *IL-1α*, (**H**) *IL-1β*, and (**I**) *IL-10*. SJE, *Sophora japonica* extract; *HO-1*, heme oxygenase-1; *NQO-1*, NAD(P)H quinone oxidoreductase 1; *GCLC*, glutamate–cysteine ligase catalytic subunit; *GCLM*, glutamate–cysteine ligase modifier subunit; *TNF-α*, tumor necrosis factor-α; *IL-6*, Interleukin-6; *IL-1α*, Interleukin-1α; *IL-1β*, Interleukin-1β; *IL-10*, Interleukin-10; Ctrl group, piglets fed the basal diet; SJE group, piglets fed the basal diet supplemented with 0.1% SJE. Data are presented as mean ± SD (*n* = 7); significance is shown as * *p* < 0.05 and ** *p* < 0.01.

The mRNA expression levels of inflammatory cytokines and antioxidant-related genes in jejunal tissue are shown in Figure 3. Compared with the Ctrl group, SJE supplementation significantly increased the mRNA expression of *HO-1* (Figure 3A, *p* < 0.01), *GCLC* (Figure 3C, *p* < 0.01), and *GCLM* (Figure 3D, *p* < 0.05) in the jejunum. Similarly, SJE supplementation significantly increased the mRNA level of the anti-inflammatory cytokine *IL-10* (Figure 3I, *p* < 0.01) compared with the Ctrl group. Overall, these results indicate that SJE enhanced antioxidant capacity and alleviated inflammation in the jejunum of early-weaned piglets.

### 3.6. SJE Supplementation Shifted Gut Microbial Structure and Diversity in Early-Weaned Piglets

**Figure 4:**
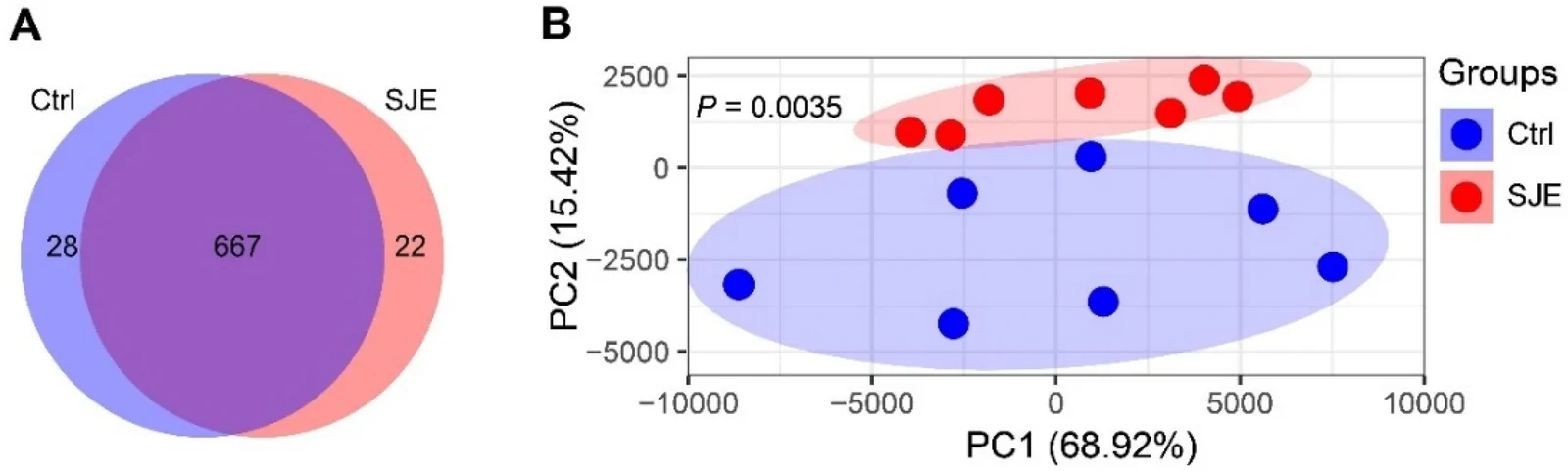
Effects of SJE supplementation on gut microbial diversity in early-weaned piglets: (**A**) Venn diagram of OTUs; (**B**) PCoA plot based on Bray–Curtis distances between groups. SJE, *Sophora japonica* extract; Ctrl group, piglets fed the basal diet; SJE group, piglets fed the basal diet supplemented with 0.1% SJE. Data are presented as mean ± SD (*n* = 7).

As shown in Figure 4, the distribution of OTUs differed between the Ctrl and SJE groups (Figure 4A), indicating distinct gut microbial community structures. PCoA based on Bray–Curtis distances showed a clear separation between the gut microbiota of the Ctrl and SJE groups (Figure 4B), and PERMANOVA confirmed a significant difference in microbial composition (*p* = 0.0035). SJE supplementation significantly increased alpha diversity indices, including observed species (*p* < 0.01), ACE (*p* < 0.05), and Chao1 (*p* < 0.05), whereas no significant differences were observed in the Shannon, Simpson, or coverage indices between groups (Table 3).

### 3.7. SJE Supplementation Shifted the Relative Abundance of Gut Microbiota in Early-Weaned Piglets

**Figure 5:**
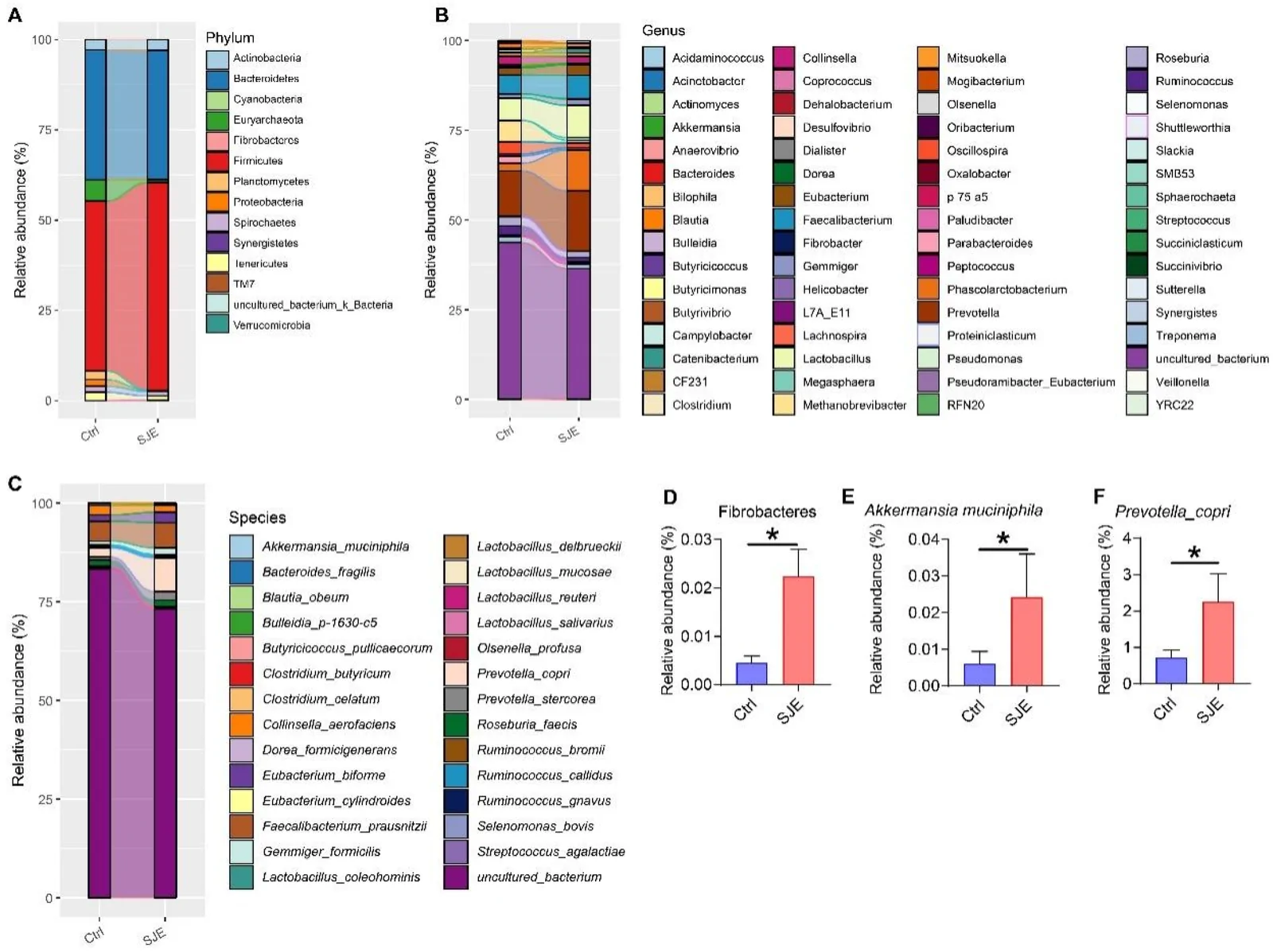
Effects of SJE supplementation on gut microbiota composition in early-weaned piglets. Relative abundances at (**A**) phylum, (**B**) genus, and (**C**) species levels. (**D**–**I**) Bacterial taxa with significant differences among groups, including Fibrobacteres (**D**), *Akkermansia muciniphila* (**E**), and *Prevotella copri* (**F**). SJE, *Sophora japonica* extract; Ctrl group, piglets fed the basal diet; SJE group, piglets fed the basal diet supplemented with 0.1% SJE. Data are presented as mean ± SD. Significance is shown as * *p* < 0.05.

The linked bar plots of the taxon abundance show that SJE supplementation markedly altered the relative abundance of bacteria at different taxonomic levels, including the phylum (Figure 5A), genus (Figure 5B), and species (Figure 5C) levels. Compared with the Ctrl, SJE supplementation increased the relative abundances of Fibrobacteres (Figure 5D, *p* < 0.05), *Akkermansia muciniphila* (Figure 5E, *p* < 0.05), and *Prevotella copri* (Figure 5F, *p* < 0.05).

### 3.8. SJE-Induced Gut Microbiota Shifts Are Significantly Correlated with Early Weaning–Related Indices

**Figure 6:**
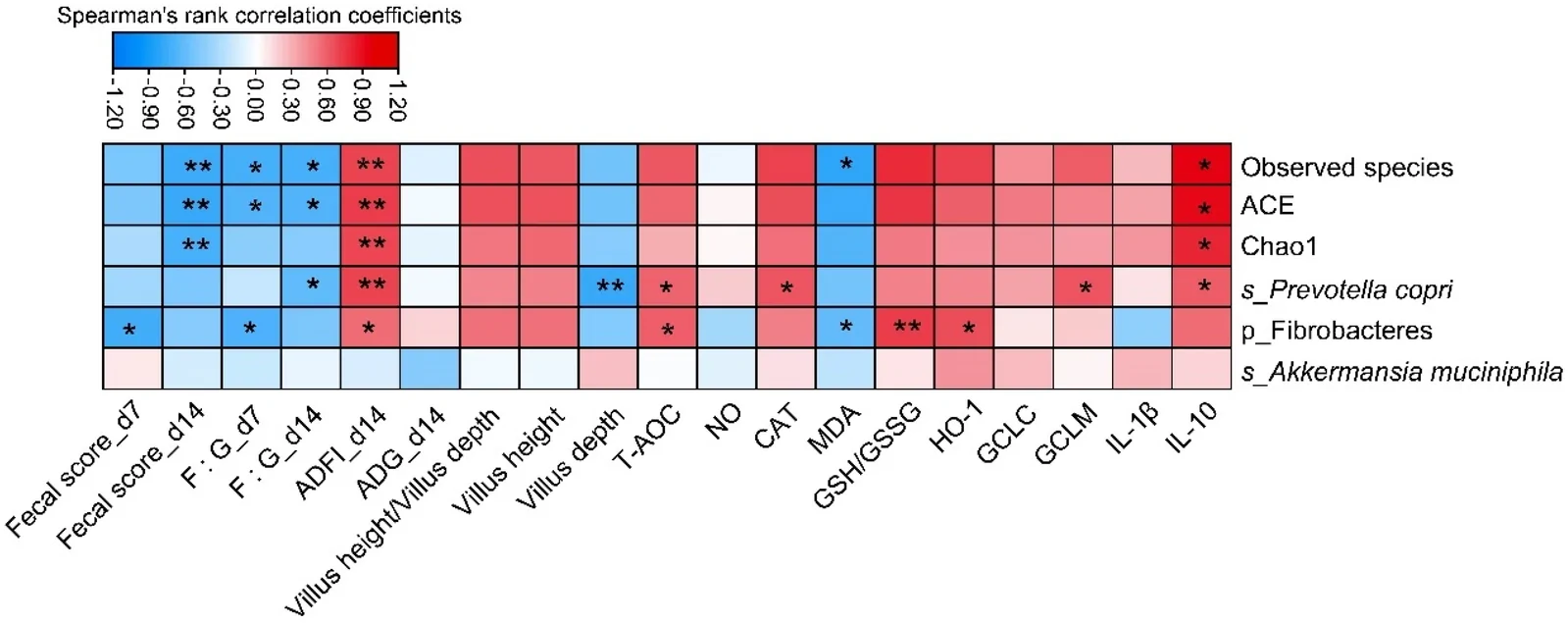
Heat map of Spearman’s rank correlation coefficients between SJE-responsive bacteria and early-weaning indices. Blue indicates a negative correlation, while red indicates a positive correlation. ACE, Abundance-Based Coverage Estimator; ADFI, average daily feed intake; ADG, average daily weight gain; F:G ratio, ratio of feed to gain; d 7, day 7 post-weaning; d 14, day 14 post-weaning; *HO-1*, heme oxygenase-1; *GCLC*, glutamate–cysteine ligase catalytic subunit; *GCLM*, glutamate–cysteine ligase modifier subunit; *IL-1β,* Interleukin-1β; *IL-10*, Interleukin-10; significance is shown as * *p* < 0.05, ** *p* < 0.01.

Spearman’s rank correlation analysis (Figure 6) revealed that the observed species index of alpha diversity was negatively correlated with fecal score on day 14 (*p* < 0.01), with the F/G ratio on day 7 (*p* < 0.05), with the F/G ratio on day 14 (*p* < 0.05), and with MDA (*p* < 0.05), while it was positively correlated with ADFI on day 14 (*p* < 0.01) and with *IL-10* (*p* < 0.05). Similarly, ACE was negatively correlated with fecal score on day 14 post-weaning (*p* < 0.01), with the F/G ratio on day 7 (*p* < 0.05), and with the F/G ratio on day 14 (*p* < 0.05), and positively correlated with ADFI on day 14 (*p* < 0.01) and with *IL-10* (*p* < 0.05). Chao1 was negatively correlated with fecal score on day 14 post-weaning (*p* < 0.01) and positively correlated with ADFI on day 14 (*p* < 0.01) and with *IL-10* (*p* < 0.05). *Prevotella copri* was negatively correlated with the F/G ratio on day 7 (*p* < 0.05) and with villus depth (*p* < 0.01), and positively correlated with ADFI on day 14 (*p* < 0.01), and with T-AOC (*p* < 0.05), CAT (*p* < 0.05), *GCLM* (*p* < 0.05), and *IL-10* (*p* < 0.05). Fibrobacteres was negatively correlated with fecal score on day 7 (*p* < 0.05), with the F/G ratio on day 7 (*p* < 0.05), and with MDA (*p* < 0.05), and positively correlated with ADFI on day 14 (*p* < 0.05), and with T-AOC (*p* < 0.05), the GSH/GSSG ratio (*p* < 0.05), and *HO-1* (*p* < 0.05).

## 4. Discussion

In the present study, dietary supplementation with 0.1% SJE significantly attenuated diarrhea in early-weaned piglets on days 7 and 14 post-weaning. These results compare well with previous research, as SJE has been reported to decrease both total and wet fecal production in a dose-dependent manner in a castor oil–induced diarrhea model in rats [17]. Wang et al. found that SJE has promising potential for the treatment of diarrhea through its anti-inflammatory effects, mediated by the inhibition of NF-κB activation [29]. Moreover, SJE markedly delayed the onset of castor oil–induced diarrhea in rats, highlighting its potential as a promising natural resource for the prevention of diarrhea [17]. Early-weaning stress typically impairs growth performance due to transient anorexia, severe intestinal damage, intestinal oxidative stress, and intestinal inflammation [4,5]. In this study, SJE supplementation improved growth performance in early-weaned piglets, as evidenced by the decreased F/G ratio on days 7 and 14, and increased BW, ADFI, and ADG on day 14. This improvement in growth performance may be attributed to the amelioration of intestinal morphology and function in SJE-supplemented piglets.

In addition, SJE has been shown to alleviate ulcerative colitis in dextran sodium sulfate–induced colitis mouse models [30]. Early-weaning stress in piglets induces intestinal oxidative stress, characterized by an imbalance between free radical production and the scavenging capacity of the antioxidant defense system [2,31,32]. Dietary SJE supplementation alleviated intestinal redox imbalance in early-weaned piglets by increasing antioxidant indices, including GSH, the GSH/GSSG ratio, T-AOC, and CAT, while reducing the oxidative metabolite MDA and GSSG in jejunal tissue. Consistently, SJE supplementation enhanced antioxidant capacity by upregulating the mRNA expression of *HO-1*, *GCLC*, and *GCLM* in the jejunum of early-weaned piglets. Moreover, SJE supplementation increased the mRNA expression levels of the anti-inflammatory cytokine *IL-10* and decreased that of the pro-inflammatory cytokine *TNF-α*. The Nrf2 pathway plays a critical role in maintaining intestinal integrity by regulating the production of pro-inflammatory cytokines and inducing the production of antioxidant enzymes [33]. Under oxidative stress, Nrf2 translocates to the nucleus and protects cells by inducing the expression of various antioxidant genes, such as *HO-1* and *NQO1* [34]. Our results showed that dietary SJE supplementation increased the mRNA expression of *HO-1*, *GCLC*, and *GCLM* in jejunal tissue. Glutamate cysteine ligase (GCL) catalyzes the first and rate-limiting step in the synthesis of the cellular antioxidant GSH [35]. Specifically, SJE supplementation upregulated the mRNA expression of *GCLC* and *GCLM*, which encode the catalytic and regulatory subunits of GCL, respectively [36]. Notably, these increases in antioxidant gene expression were consistent with the elevated antioxidant indices observed in jejunal tissue. Consistent with our findings, SJE has been reported to alleviate diarrhea through its anti-inflammatory effects mediated by inhibition of NF-κB activation in an *E. coli*-challenged mouse model [29]. Overall, SJE enhanced the antioxidative capacity and attenuated the inflammation in the jejunal tissue of early-weaned piglets.

Mechanistically, oxidative stress in the inflamed intestinal mucosa disturbs the oxygen-sensitive niches occupied by the microbiota, thereby triggering gut microbiota dysbiosis [2,11]. A previous study reported that SJE could regulate chemically induced murine colitis by targeting the NF-κB signaling pathway and modulating the gut microbiota [37]. In the present study, 16S rDNA sequencing revealed that dietary SJE supplementation shifted the gut microbial structure and increased alpha diversity indices, including observed species, ACE, and Chao1. Specifically, SJE supplementation increased the relative abundances of short-chain fatty acid–producing bacteria, such as Fibrobacteres and *Prevotella copri* [38,39], as well as the anti-inflammatory bacterium *Akkermansia muciniphila* [40]. Short-chain fatty acids produced by the gut microbiota play a crucial role in maintaining intestinal homeostasis [41]. For instance, propionic acid can improve intestinal barrier function, inflammation, and oxidative stress via the signal transducer and activator of transcription 3 signaling pathway [42]. Furthermore, Spearman’s rank correlation coefficient and significance testing revealed that the identified SJE-responsive bacteria were significantly correlated with the examined early weaning–related indices, indicating that the gut microbiota contributed to the beneficial effects of SJE on alleviating intestinal oxidative stress, inflammation, and microbiota dysbiosis in early-weaned piglets.

## Figures and Tables

**Table 1:** Effects of SJE supplementation on the fecal scores of early-weaned piglets.

Item	Ctrl	0.05% SJE	0.1% SJE
Fecal scores			
d 7 post-weaning	3.00 ± 0.65 ^a^	2.63 ± 0.71 ^ab^	1.83 ± 0.47 ^b^
d 14 post-weaning	2.50 ± 0.58 ^a^	2.13 ± 0.65 ^ab^	1.50 ± 0.41 ^b^

Note: d, day; SJE, *Sophora japonica* extract; Ctrl group, piglets fed the basal diet; 0.05% SJE group, piglets fed the basal diet supplemented with 0.05% SJE; 0.1% SJE group, piglets fed the basal diet supplemented with 0.1% SJE. Data are presented as pen mean ± SD (*n* = 7). ^a,b^ Within each row, values with a different superscript letter are significantly different (*p* < 0.05).

**Table 2:** Effects of SJE supplementation on growth performance in early-weaned piglets.

Item	Ctrl	SJE	*p*-Value
days 0 to 7			
BW at d 0, kg	6.35 ± 0.46	6.39 ± 0.39	0.88
BW at d 7, kg	6.94 ± 0.45	7.04 ± 0.32	0.68
ADFI, g	145.59 ± 24.91	149.24 ± 25.88	0.81
ADG, g	84.50 ± 18.20	92.82 ± 24.31	0.48
F:G ratio	2.13 ± 0.06	1.91 ± 0.14	0.02
days 8 to 14			
BW at d 14, kg	7.67 ± 0.50	8.28 ± 0.35	0.029
ADFI, g	171.95 ± 17.06	201.39 ± 18.33	0.019
ADG, g	103.31 ± 22.70	160.88 ± 34.49	0.0012
F:G ratio	1.74 ± 0.28	1.44 ± 0.18	0.046

Note: ADFI, average daily feed intake; ADG, average daily weight gain; BW, body weight; F:G ratio, ratio of feed to gain; SJE, *Sophora japonica* extract; d 0, the day of weaning; d 7, day 7 post-weaning; d 14, day 14 post-weaning. Ctrl group, piglets fed the basal diet; SJE group, piglets fed the basal diet supplemented with 0.1% SJE. Data are presented as pen mean ± SD (*n* = 7).

**Table 3:** Effects of SJE supplementation on gut microbial diversity in early-weaned piglets.

Item	Ctrl	SJE	*p*-Value
Alpha diversity			
Observed species	447.83 ± 30.23	501.00 ± 19.56	0.0049
ACE	496.46 ± 30.52	535.97 ± 11.00	0.014
Chao1	505.09 ± 35.55	543.30 ± 14.31	0.035
Simpson	0.96 ± 0.013	0.96 ± 0.017	0.45
Shannon	6.02 ± 0.46	6.18 ± 0.48	0.59
Coverage	0.99 ± 0.0001	0.99 ± 0.0001	0.069

Note: SJE, *Sophora japonica* extract; ACE, Abundance-Based Coverage Estimator; Ctrl group, piglets fed the basal diet; SJE group, piglets fed the basal diet supplemented with 0.1% SJE. Data are presented as pen mean ± SD (*n* = 7).

## Data Availability

The original contributions presented in this study are included in the article/Supplementary Materials. Further inquiries can be directed to the corresponding authors.

## Supplementary Materials

The following supporting information can be downloaded at: https://www.mdpi.com/article/10.3390/vetsci13090887/s1, Table S1. Ingredients and nutrients levels of basal diets (as-fed basis, %). Table S2. Primers sequences of target genes selected for analysis by real-time.

## Abbreviations

The following abbreviations are used in this manuscript:

SJE	*Sophora japonica* extract
ADG	Average daily gain
ADFI	Average daily feed intake
T-AOC	Total antioxidant capacity
CAT	Catalase
MDA	Malondialdehyde
GSH	Glutathione
NO	Nitric oxide
GSSG	Glutathione disulfide
BW	Body weight
